# Caesarean Scar Ectopic Pregnancy in Early Gestation: A Scoping Review of Definitions and Diagnostic Approach

**DOI:** 10.1111/1471-0528.70122

**Published:** 2025-12-25

**Authors:** Simrit Nijjar, Lucrezia V. De Braud, Ewelina Rogozińska, Cecilia Bottomley, Davor Jurkovic, Eric Jauniaux

**Affiliations:** ^1^ EGA Institute for Women's Health, Faculty of Population Health Sciences University College London (UCL) London UK; ^2^ The EVIE Evidence Synthesis Research Group, Institute of Clinical Trials and Methodology, Faculty of Population Health Sciences University College London London UK

**Keywords:** caesarean scar ectopic pregnancy, classification, definition, diagnosis, magnetic resonance imaging, reference standard, ultrasound imaging

## Abstract

**Background:**

Caesarean scar ectopic pregnancy (CSEP) is defined by the implantation and development of a gestational sac inside a caesarean scar defect, but variations in classification systems and diagnostic criteria exist.

**Objectives:**

This study aimed to systematically review the different criteria used in the medical literature to diagnose CSEP in the first trimester of pregnancy.

**Search Strategy:**

Systematic search of PubMed, MEDLINE and Google Scholar from September 1990 to January 2024.

**Selection Criteria:**

We included prospective and retrospective observational studies published in English reporting on imaging criteria used to diagnose CSEP.

**Data Collection and Analysis:**

Two reviewers independently reviewed retrieved articles and performed data extraction using a priori‐developed data collection form. Findings were tabulated and synthesised in a narrative format.

**Main Results:**

A total of 22 studies, involving 1749 CSEP cases, met the inclusion criteria. Diagnostic modalities varied, with eight different classification systems reported across 11 studies, with the remaining 11 studies not specifying a classification system. Histology was used as a reference standard in 59% (13/22), intraoperative features in 18% (4/22) and a combination of both in 23% of studies (5/22). Seventy‐two percent of studies (13/18) that used histology as a reference standard did not provide specific histological criteria for diagnosing CSEP.

**Conclusions:**

This review highlights the wide variability in diagnostic approaches, imaging criteria and classification systems used in the first‐trimester diagnosis of CSEP. The absence of a universally accepted reference standard for CSEP diagnosis poses a major challenge for prospective studies evaluating diagnostic accuracy.

## 1 Introduction

Caesarean scar ectopic pregnancy (CSEP) is defined as the implantation of the blastocyst and the development of the gestational sac within a myometrial scar defect following one or more previous caesarean births (CBs) [1]. First described by Larsen et al. in 1978 [2], CSEP was not formally recognised as a variant of uterine ectopic pregnancy by the international gynaecological community until 2020 [3]. CSEP is associated with a high risk of maternal morbidity and mortality [4], hence the importance of early recognition and timely management. It has been demonstrated that diagnosing and treating CSEP before 9 weeks of gestation markedly reduces the risk of maternal complications [5]. However, there remains an ongoing terminology debate and lack of consensus in distinguishing between a pregnancy implanted inside a caesarean scar defect (CSD) and one implanted low in the uterine cavity near or at the scar, but not within the defect or on top of a well‐healed scar [5]. This lack of uniformity in defining CSEP complicates the analysis and comparison of findings across studies focused on the epidemiology, diagnosis and management of CSEP. Key terms and definitions used throughout this review are summarised in Box 1.

BOX 1 **Key definitions.**

**Table:** 

Term	Definition
CSEP/CSP (Caesarean Scar Ectopic Pregnancy/Caesarean Scar Pregnancy)	Synonymous terms referring to a pregnancy implanted within the myometrial defect caused by dehiscence of the anterior lower uterine segment caesarean scar. ‘CSEP’ explicitly denotes that this implantation is in an ectopic location, but both terms are used interchangeably in the literature. There remains ongoing debate as to whether such implantations should be classified as ectopic or not
CSD (Caesarean Scar Defect)/Niche	A localised indentation at the site of a previous caesarean section scar on the anterior lower uterine wall, typically defined as a depth of ≥ 2 mm on ultrasound
RMT (Residual Myometrial Thickness)	The thickness of remaining myometrium between the gestational sac (or defect) and the uterine serosa, measurable by ultrasound; thought to be a key indicator of rupture risk
CDI (Colour Doppler Imaging)	Ultrasound technique assessing vascularity around the gestational sac to confirm implantation site and reduce misclassification. Perigestational blood flow can be assessed semi‐quantitatively using a 1–4 vascularity scale, where higher grades indicate more extensive circumferential flow
Placental lacunae	Large, irregular, fluid‐filled vascular spaces within placental tissue, often containing visibly turbulent flow on ultrasound. Their presence or absence can assist in assessing abnormal placentation in CSEP
Endogenic (Type 1)/Exogenic (Type 2)	Patterns of CSEP implantation. A Type 1 CSEP (endogenic type) is characterised by more than 50% of the gestational sac protruding into the uterine cavity, whereas a Type 2 CSEP (exogenic type) has less than 50% of the sac extending into the cavity, with growth directed outward toward the bladder or peritoneal cavity
Reference standard	The benchmark diagnostic method or combination of findings used to confirm a diagnosis, against which other tests or imaging criteria are compared (e.g., histopathology or intraoperative visual confirmation for CSEP)

The reported incidence of CSEP ranges from 1 in 1800 to 1 in 2200 pregnancies, although these figures are likely underestimates due to misdiagnosis and underreporting [6]. The incidence is expected to rise alongside the increasing global prevalence of CB [7]. The first ultrasound‐based diagnosis of CSEP was described by Rempen and Albert in 1990 [8]. Since then, numerous sonographic and magnetic resonance imaging (MRI) diagnostic criteria have been proposed. These criteria include classifications such as ‘on the scar’ versus ‘in the niche’ and distinctions based on the gestational sac extension into the uterine cavity, including ‘endogenic/type 1’ versus ‘exogenic/type 2’ implantations [8, 9]. More recently, Jordans et al. [10] proposed a new sonographic classification system, categorising CSEPs into three types based on the position of the gestational sac relative to the uterine cavity and serosal line. Several studies have explored the use of MRI as an additional imaging tool to ultrasound imaging when the diagnosis remains inconclusive [11, 12, 13, 14, 15, 16, 17, 18, 19]. Both imaging techniques are operator dependent. By contrast, compared to ultrasound imaging, no distinct MRI diagnostic criteria for CSEP have been formally proposed and radiologists often apply the same criteria established for ultrasound assessment.

To address these issues, we conducted a scoping review to systematically examine the existing literature and identify key knowledge gaps. This review focuses on the diagnostic criteria, classification systems and imaging modalities used in diagnosing CSEP during the first trimester.

## 2 Methods

### 2.1 Study Protocol

The study was guided following a prospectively developed protocol registered with PROSPERO (CRD42022375897). The review is reported in line with the Preferred Reporting Items for Systematic Reviews and Meta‐Analyses extension for Scoping Review (PRISMA‐ScR) [20] and the Arksey and O'Malley methodological framework for scoping reviews [21].

### 2.2 Search Strategy and Study Selection

We systematically searched PubMed (National Library of Medicine platform), MEDLINE (Ovid) and Google Scholar to identify relevant articles published in the international literature between the publication of Rempen and Albert in 1990—the first description of CSEP diagnosed by ultrasound—and January 2024.

The following terms were used: ‘caesarean scar ectopic pregnancy’ OR ‘caesarean scar pregnancy’ AND ‘diagnosis’ OR ‘classification’ OR ‘definition’ OR ‘ultrasonography’ OR ‘transabdominal’ OR ‘transvaginal’ OR ‘MRI’. Search strategies were iteratively refined to ensure inclusion of variant terminology and spelling, and search fields were adapted to the functionality of each database.

Full search strategies for each database are provided in Table S1. Reference lists of all included articles and relevant reviews were manually screened to identify additional studies. Two reviewers (S.N. and L.D.B.) independently assessed identified titles and abstracts against the eligibility criteria, with disagreements resolved by discussion with a third investigator (C.B.).

### 2.3 Inclusion and Exclusion Criteria

The inclusion criteria were observational studies with a sample size of 10 or more women diagnosed with CSEP in the first trimester using ultrasound or MRI. Eligible studies had to report both the diagnostic criteria and the ‘reference standard’ used by the authors and to be published in English. Exclusion criteria included non‐English language articles, abstracts only, letters to the editor, studies where ultrasound or MRI were not used for diagnosing CSEP, and studies where CSEP was diagnosed after 12 weeks of gestation.

### 2.4 Data Extraction Process

Two investigators (S.N. and L.D.B.) independently assessed the content of the full text articles using a predefined Excel spreadsheet and extracted the following study characteristics and outcome data: first author, publication year, country of origin, study design, sample size, gestational age, method of diagnosis, reference standard reported by the study authors, definitions and diagnostic criteria used and diagnostic accuracy data if available.

### 2.5 Data Synthesis

A descriptive analytical approach was adopted. Extracted data on study characteristics and diagnostic criteria were tabulated and synthesised narratively. Simple descriptive summaries of reported proportions of CSEP within the included studies were produced to illustrate patterns in the data. Given the heterogeneity of study designs and populations, no formal meta‐analysis or quantitative pooling was undertaken, and the results are presented as descriptive frequencies only. The formal evaluation of diagnostic accuracy of CSEP criteria was not possible due to the heterogeneity of the available data.

## 3 Results

### 3.1 Included Studies

**FIGURE 1:**
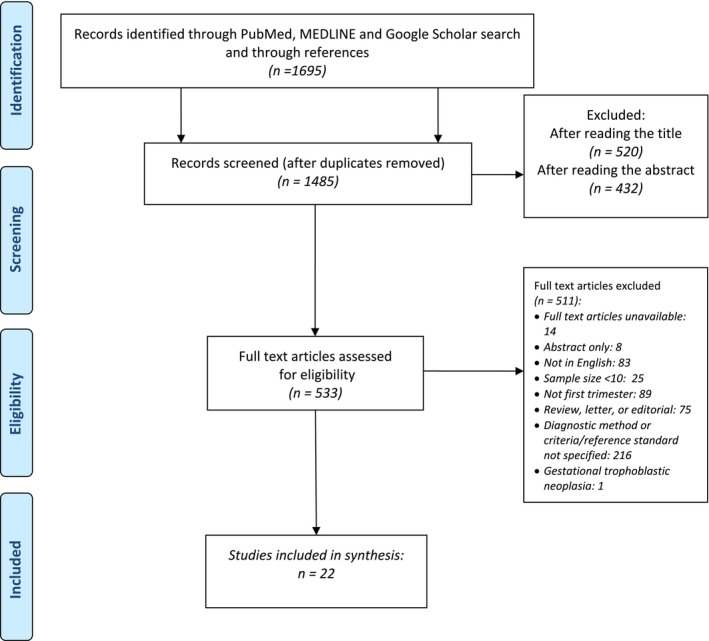
Flowchart showing studies identified through literature search (01.01.1990 to 01.01.2024).

The study selection process is detailed in Figure 1. A total of 22 studies met the inclusion criteria (Table 1) [8, 22, 23, 24, 25, 26, 27, 28, 29, 30, 31, 32, 33, 34, 35, 36, 37, 38, 39, 40, 41, 42], collectively reporting on 1749 participants with CSEP.

**TABLE 1:** Characteristics of studies included in the scoping review.

Author, year	Journal (1 = specialist, 2 = general)	Country	Study design	Single centre (Y/N)	Study period	Total sample size (*n*)	No. of CSEP cases (*n*)	Percentage of CSEP cases (%)	Gestation at diagnosis (days)^a^	Modality used for diagnosis	‘Reference standard’ for confirmation of diagnosis
Wang 2013	2	China	Retrospective case series	Y	03/2008–11/2011	11	11	100	46–78	USS & doppler	Intraoperative features
Zhang 2013	1	China	Retrospective case series	Y	06/2005–03/2011	10	10	100	< 91	USS & office hysteroscopy	Histology
Huang 2014	1	China	Retrospective cohort	Y	05/2010–09/2013	42	42	100	< 91^b^	TAS & TVS vs. Gadolinium enhanced MRI	Histology
Liu 2014	2	China	Retrospective cohort	Y	08/2012–06/2014	81	68	84	30–84	TVS vs. MRI	Histology
Timor 2014	1	USA, Italy	Prospective cohort	N	2 years	10	10	100	39–65	USS	Histology
Qian 2015	1	China	Prospective cohort	Y	02/2008–01/2013	66	66	100	≤ 70	TVS	Histology
Xiong 2016	1	China	Prospective cohort	Y	03/2014–03/2015	92	52	57	49 (35–77)	USS vs. CEUS	Intraoperative features
Cali 2017	1	Italy	Retrospective case series	Y	01/2007–12/2015	68	68	100	42–56	USS	Histology
Kaelin Agten 2017	1	USA, Italy	Retrospective cohort	N	2013–2015	17	17	100	56 (35–63)	TVS	Histology^c^
Qian 2017	1	China	Retrospective cohort	Y	03/2011–09/2014	45	45	100	≤ 84	TVS	Histology & Intraoperative features
Qian, Weng 2017	1	China	Prospective case–control	Y	10/2013–09/2014	40	20	50	< 70	USS	Histology
Wu 2019	2	China	Prospective cohort	Y	01/2017–03/2018	485	220	45	< 77	TVS vs. CEUS	Histology & Intraoperative features
Li 2020	1	China	Retrospective cohort	Y	01/2016–02/2019	30	27	90	57.73 ± 15.1	TVS vs. CEUS	Histology
Li, Dai 2020	2	China	Prospective cohort	Y	01/2011–05/2018	93	66	71	35–69	TVS & doppler	Intraoperative features^d^
Liu 2020	2	China	Retrospective cohort	Y	12/2017–12/2018	35	24	69	46.34 ± 8.82	CEUS	Histology & Intraoperative features
Aslan 2021	1	Turkey	Retrospective cohort	Y	01/2015–04/2019	42	42	100	35–67	TVS	Histology
Shi 2021	2	China	Prospective cohort	Y	01/2017–12/2019	142	97	68	≤ 84	TVS & 3‐D vs. TVS & 3‐D & doppler	Histology & Intraoperative features
Tang 2021	1	China	Retrospective cohort	Y	01/2013–06/2018	439	439	100	< 91	USS	Histology
Huang 2022	2	China	Retrospective cohort	N	06/2015–11/2021	54	54	100	35–84	TVS & 3‐D & doppler vs. MRI	Histology & Intraoperative features
Wang 2022	2	China	Retrospective cohort	Y	11/2019–11/2020	65	54	83	28–63	TAS vs. TVS vs. MRI	Histology
Cai 2023	2	China	Retrospective cohort	Y	01/2010–12/2021	385	252	65	< 91	TVS	Intraoperative features
Feng 2024	2	China	Retrospective cohort	Y	01/2022–01/2023	65	65	100	37–62	2‐D TVS & TAS vs. 3‐D TVS & TAS vs. 2‐D/3‐D TVS & TAS	Histology

Abbreviations: 3‐D, three dimensional; CEUS, contrast‐enhanced ultrasound; MRI, magnetic resonance imaging; TAS, transabdominal ultrasound; TVS, transvaginal ultrasound; USS, ultrasound (not defined as TVS or TAS).

^a^ As reported in the study.

^b^ Information not reported in study, contacted author.

^c^ Only available for 11 of the 17 cases, as other cases did not undergo caesarean hysterectomy.

^d^ Only available for 49 of the 66 cases, as other cases underwent dilatation and curettage.

### 3.2 Study Characteristics

The study characteristics are presented in Table 1. There were three case series (14%) [22, 23, 29], 18 cohort studies (82%) [8, 24, 25, 26, 27, 28, 30, 32, 33, 34, 35, 36, 37, 38, 39, 40, 41, 42] and one case–control study (5%) [31]. Of these, only seven (32%) [26, 27, 28, 31, 32, 34, 37] employed a prospective design, and no randomised controlled trials (RCTs) were identified. Regarding publication outlets, 12 studies (55%) [8, 23, 24, 26, 27, 28, 29, 30, 31, 33, 36, 38] appeared in specialist obstetrics and gynaecology journals, while ten studies (45%) [22, 25, 32, 34, 35, 37, 39, 40, 41, 42] were published in general medical journals. Geographically, 18 of the selected studies (82%) [22, 23, 24, 25, 27, 28, 30, 31, 32, 33, 34, 35, 37, 38, 39, 40, 41, 42] originated from the People's Republic of China, with the remaining studies conducted in the United States or Italy (three studies, 14%) [8, 26, 29] and Turkey (one study, 5%) [36]. Nineteen studies [22, 23, 24, 25, 26, 27, 28, 29, 30, 31, 32, 33, 34, 35, 36, 37, 38, 39, 40, 41, 42] were conducted at single institutions, with only three (14%) [8, 26, 39] involving multiple institutions. The median sample size of CSEP cases in the included studies was 53 participants (range: 10–439). Across the included studies, the proportion of reported pregnancies classified as CSEP ranged widely, but on average represented approximately 92% of examined cases. Considerable variation was observed between studies (*I* ^2^ = 97.7%), consistent with heterogeneity in study design and diagnostic inclusion criteria.

Among 22 studies analysed, 17 (77%) reported varied CSEP treatments. Approaches included suction evacuation alone [25, 31] or combined with uterine artery embolisation (UAE) [25, 28, 33], Foley balloon insertion [36], hysteroscopic resection with or without UAE [24, 27, 38] or methotrexate [38]. Surgical options ranged from hysterectomy [36] or caesarean hysterectomy [8, 26, 29], resection of the CSD [25] and removal of the pregnancy via hysteroscopic, laparoscopic, or open resection [33]. Combined approaches were also described. Notably, five studies (23%) [32, 37, 39, 40, 42] did not specify the treatments utilised for managing CSEP, despite referencing histological findings or intraoperative features to confirm CSEP diagnosis.

Several imaging modalities, alone or in combination, were used to diagnose CSEP, including non‐specified ultrasound (7/22, 32%) [22, 23, 26, 28, 29, 31, 38], transvaginal scan (14/22, 64%) [8, 24, 25, 27, 30, 32, 33, 34, 36, 37, 39, 40, 41, 42], transabdominal scan (3/22, 14%) [24, 40, 42], contrast‐enhanced ultrasound (4/22, 18%) [28, 32, 33, 35] and MRI (4/22, 18%) [24, 25, 39, 40]. The diagnostic criteria used are described below.

### 3.3 Diagnostic Criteria

Across all 22 studies, common diagnostic signs included a gestational sac developing within or near the CSD, an empty uterine cavity and cervical canal and a thin or absent myometrial layer between the gestational sac and the bladder (Table S1). Seventeen studies (77%) classified CSEP as an ectopic pregnancy [22, 23, 25, 27, 28, 29, 30, 31, 32, 34, 35, 36, 37, 38, 39, 40, 41], five studies (23%) did not explicitly refer to it as such [8, 24, 26, 33, 42]. Fourteen studies (64%) defined the implantation site as ‘in the scar’ [22, 23, 27, 28, 29, 30, 31, 34, 35, 36, 37, 38, 41, 42], while six studies (27%) described it as ‘at the scar site’, ‘in the region of the scar’, or ‘in the lower anterior uterine segment’ [24, 25, 32, 33, 39, 40]. The remaining two studies (9%) used definitions such as ‘in the scar’ or ‘on the scar’ [8, 26].

For the purpose of this review, ‘in the scar’ or ‘in the niche’ refers to implantation within a CSD, while ‘on the scar’ or ‘near the scar’, indicate implantation adjacent to, but not within, the CSD. The included studies primarily examined pregnancies implanted in the lower uterine segment scar, although cases involving classical (upper segment) scars were also eligible. This distinction is clinically relevant, as implantation in a classical scar may be misclassified as an intramural pregnancy (IMP), which has different management implications. While continuation of IMPs is generally not recommended, selected CSEPs can, after appropriate counselling, be managed expectantly and may result in a live birth. Clarifying this distinction may improve diagnostic accuracy and guide more individualised management decisions.

Ultrasound was the primary diagnostic tool across all studies (Table 1), and 14 studies (64%) reported whether the pregnancy was a live or failed CSEP [8, 22, 25, 26, 27, 29, 33, 35, 36, 37, 38, 39, 40, 41]. Ultrasound criteria for evaluating the gestational sac included its location, morphology and vascularity. Colour Doppler imaging (CDI) was used in 16 studies (73%) to assess vascularity at the implantation site [8, 22, 26, 28, 29, 32, 33, 34, 35, 36, 37, 38, 39, 40, 41, 42]. No studies reported on placental lacunae. Two authors reported additional ultrasound findings, such as the ‘organ sliding sign’ [37] and precise measurements of gestational sac dimensions relative to anatomical landmarks [41]. Detailed diagnostic definitions are presented in Table S2. A comparative summary of the eight published classification systems, using standardised terminology, is shown in Table 2.

**TABLE 2:** Comparison of published classification systems for CSEP and their conceptual alignment with the Endogenic–Exogenic framework.

Classification type	Defining parameters	Corresponding terminology	Key features
Type 1–2	Type 1: implantation within the scar growing toward the uterine cavity Type 2: implantation deeply in the scar growing outward toward the serosa or bladder	Type 1 = Endogenic; Type 2 = Exogenic	Based on direction of gestational sac growth relative to the uterine cavity and serosa
Endogenic/Exogenic system	Endogenic: implantation on scar surface extending into uterine cavity Exogenic: implantation deeply in scar, growing outward toward bladder	Endogenic = Type 1; Exogenic = Type 2	Classification based on direction of implantation—into the cavity (endogenic) versus outward toward the serosa or bladder (exogenic)
On‐scar/In‐niche system	On‐scar: implantation on top of a well‐healed scar In‐niche: implantation within a deficient scar niche	On‐scar = Endogenic; In‐niche = Exogenic	Distinguishes implantation on a well‐healed scar from implantation within a dehiscent scar
Gestational‐sac/Mixed‐mass system	Gestational‐sac type: discrete sac‐like structure at the scar Mixed‐mass type: heterogeneous mass with indistinct margins	Sac‐type = Endogenic; Mass‐type = Exogenic	Morphology and echogenicity of the pregnancy within the scar
Incision gestational‐sac/Mass‐bulk system	Incision‐sac type: intact sac within scar Mass‐bulk type: solid or mixed‐echo lesion replacing the sac	Endogenic/Exogenic correspondence unclear	Morphology‐based description focusing on echogenic mass appearance
Type 1–3 system	Type 1: partial implantation Type 2: complete implantation confined to scar Type 3: mixed echogenic mass replacing sac morphology	Type 1 = Endogenic; Type 2 = Exogenic Type 3 correspondence unclear	Based on extent of implantation and characteristic ultrasound appearance
Type I–III (RMT‐based system)	Type I: RMT > 3 mm Type II: RMT ≤ 3 mm Type III: complete implantation bulging beyond serosa, RMT ≤ 3 mm	Type I = Endogenic; Type II–III = Exogenic	Based on RMT and implantation depth
Unruptured/Mass‐bulk descriptors	Unruptured type: gestational tissue confined to scar site without rupture overlaps with mass/bulk type morphology	Unruptured = Exogenic	Emphasises intact scar contour

*Note:* Corresponding terminology indicates the conceptual alignment of each system's categories with the commonly used Endogenic–Exogenic framework. Classification systems are grouped by conceptual focus (directional, anatomical, morphological, quantitative and descriptive) rather than by nomenclature. Alignment with the Endogenic–Exogenic framework was interpreted from described implantation direction or morphology where sufficient detail was available; for some systems (e.g., [37, 39]), correspondence could not be determined for all subtypes due to limited description.

Abbreviation: RMT, residual myometrial thickness.

Classification systems exhibited considerable variability, with 11 studies (50%) not classifying CSEPs into distinct types [24, 26, 27, 28, 29, 31, 33, 34, 36, 40, 41]. Among the remaining studies, eight different classification systems were reported [8, 22, 23, 25, 30, 32, 35, 37, 38, 39, 42]. Two studies used the terms ‘incision gestational sac type’, ‘mass‐bulk type’ and ‘unruptured type’ without providing explanatory definitions [30, 39]. Liu et al. classified CSEPs as ‘gestational sac type’, and ‘mixed mass type’ and defined them as a gestation sac structure or mass with unclear boundaries at or in the lower uterine scar [25]. Three authors used terms ‘endogenous/type 1’ and ‘exogenous/type 2’ to describe implantation patterns, with the former referred to implantation growing predominantly into the uterine cavity (partial implantation), and the latter referred to growth toward the abdominal cavity or bladder (complete implantation) [22, 23, 35]. Shi et al. further identified a ‘type 3 CSEP’ as a ‘mixed echogenic mass’ [37]. Alternative classifications categorised CSEPs as implanted ‘on the scar’ or ‘in the niche’ [8]. Three studies reported more structured classification systems based on residual myometrial thickness (RMT) and implantation depth, categorising CSEPs into three types depending on these parameters [32, 38, 42]. None of the studies evaluated the reproducibility of diagnostic criteria.

### 3.4 Reference Standard Criteria

Histology alone was utilised as a reference standard in 13 studies (59%) [8, 23, 24, 25, 26, 27, 29, 31, 33, 36, 38, 40, 42]. Intraoperative features (visualising the pregnancy at surgery in the scar area) served as the reference standard in four studies (18%) [22, 28, 34, 41], comprising laparoscopy (*n* = 2) [22, 34], hysteroscopy (*n* = 1) [41] and uterine curettage (*n* = 1) [28]. The remaining five studies (23%) employed both histology and surgical findings as reference standards [30, 32, 35, 37, 39].

Among the studies that used histology as a reference standard, five (28%) reported the criteria used for histological assessment [8, 23, 26, 29, 33]. These criteria varied across studies. Two studies [23, 35] reported the presence of chorionic villi and/or trophoblastic tissue within the myometrium or the scar area in cases of CSEP. Histological evidence of placenta increta or percreta was reported in two other studies, though specific diagnostic criteria used to reach these conclusions were not provided [8, 26] The final study provided more detailed criteria, diagnosing placenta accreta when anchoring villi were attached to the myometrium without invading it, placenta increta when villi penetrated the myometrium and placenta percreta when villi extended through the myometrium to the uterine serosa or adjacent organs [29].

## 4 Discussion

### 4.1 Main Findings

The analysis of the data from 22 observational studies included in our scoping review found variation in study design and substantial heterogeneity in imaging criteria used for diagnosing CESP in the first trimester. We found several methodological issues with the studies, including unclear diagnostic thresholds and a lack of a robust reference standard.

### 4.2 Strengths and Limitations

This is the first scoping review to systematically examine the different criteria used to diagnose CSEP. Our synthesis provides a structured overview of how diagnostic features have been reported across studies. Across the included literature, approximately 92% of pregnancies were classified as CSEP. This high proportion likely reflects selection bias, as most studies enrolled women already suspected or diagnosed with CSEP rather than unselected early pregnancies. This value is presented descriptively within the text, not as a pooled estimate of prevalence or diagnostic accuracy. The finding underscores the difficulty of assessing diagnostic performance when study populations predominantly include women already diagnosed or suspected of having the condition under investigation.

A key limitation of this review is that most included studies (18 of 22) originated from the People's Republic of China, which may limit the generalisability of the findings. Several studies were conducted at large tertiary centres within the same regions, and while potential overlap between centres or patient cohorts could not be confirmed, it also cannot be fully excluded. Such duplication may have contributed to the apparent consistency of certain diagnostic criteria across studies; however, when considered collectively, there remained substantial variation in diagnostic definitions, study design and patient characteristics. Importantly, no universally accepted reference standard for confirming CSEP—whether by imaging, clinical outcomes, or histopathology—was identified, precluding direct comparison between studies.

Despite these limitations, the scoping review approach allowed us to map existing evidence comprehensively, identify key gaps and highlight areas where further research is needed. The findings provide a foundation for developing standardised diagnostic criteria and imaging guidelines to improve accuracy and comparability in future studies.

### 4.3 Interpretation

Key ultrasound criteria for evaluating the gestational sac—such as location, morphology and vascularity—were inconsistently applied across studies, emphasising the need for standardised diagnostic criteria. We demonstrated that despite international imaging criteria endorsed by specialist societies [3], many studies reported CSEP in cases where the pregnancy was not implanted in the CSD but was simply located ‘low’ in the uterine cavity in patients with a history of CB. Despite CDI having been shown to reduce false positives by more accurately determining the location of the definitive placenta in relation to the CSD, over a quarter of studies did not use CDI to assess peri‐placental and intraplacental vascularity [43].

The absence of CDI in a substantial proportion of studies introduces a risk of false‐positive or misclassified diagnoses, particularly in pregnancies located close to but not within the CSD. CDI provides critical information on peritrophoblastic flow and the relationship between the gestational sac, uterine cavity and bladder wall. To reduce diagnostic variability, future research should adopt a standardised imaging dataset. Building on previous Delphi consensus recommendations [10], this review proposes that essential items to document include the gestational age at diagnosis and/or treatment; the presence or absence of cardiac activity when a fetus is visible; the location and extent of the pregnancy in relation to the CSD, including whether it is completely confined within the defect or partially protrudes into the uterine cavity, and its position relative to the cervix and uterine serosa; RMT at diagnosis and during subsequent scans; pregnancy size; CDI assessment of vascularity; presence or absence of placental lacunae; and the relationship of the pregnancy to the deep uterine vessels.

While previously proposed diagnostic markers such as the crossover sign [29] or the uterine cavity and serosal lines [10] may provide supportive information, these features are influenced by gestational age and the progressive changes in uterine anatomy as the pregnancy enlarges. Their diagnostic value may therefore be limited by temporal factors, and they cannot currently be recommended as core criteria. Consistent reporting of the core parameters listed above would improve diagnostic accuracy, reduce misclassification and enhance comparability across studies.

Standardising imaging parameters is essential, but to achieve diagnostic consistency, harmonisation of the classification frameworks applied to CSEP is also required. We identified eight different classification systems for diagnosing CSEP. While some studies employed detailed diagnostic classifications, others relied on less well‐defined descriptive approaches, contributing to variability in the methods used. Such variation in existing classification underscores the urgent need for standardised frameworks and consistent reporting practices to improve uniformity and reliability in CSEP diagnosis.

Early and accurate detection and treatment of CSEP is critical for improving patient outcomes and preventing complications associated with this condition [5, 44]. However, it is important to recognise that population‐based estimates suggest a much lower prevalence of CSEP, approximately 1.5 per 10 000 maternities [45]. This discrepancy raises concerns about the accuracy of case ascertainment in the studies included in this review. In this review, the apparent high frequency of CSEP diagnoses reported in the first trimester likely reflects both selection bias and potential overdiagnosis or misclassification, reinforcing the need for standardised, evidence‐based diagnostic protocols.

Over 70% of studies using histopathology as a reference standard did not provide any description of the criteria used for the diagnosis. Among those that did, the absence of decidua with direct attachment of villi to the myometrium or uterine serosa was reported as the main diagnostic criterion. This histologic criterion was first used by Irving and Hertig in 1937 for the diagnosis of placenta accreta at delivery in the third trimester of pregnancy [46]. However, the development of accreta placentation is a progressive phenomenon secondary to high‐velocity flow entering the intervillous space of the placenta in the second and third trimester [47]. This leads to the distortion in the uteroplacental interface with loss of parts of the physiological site of detachment of the placenta from the uterine wall [48]. Thick fibrinoid deposition at the uteroplacental interface in the accreta areas in the third‐trimester placenta indicates that there is more than the absence of decidua in the diagnosis of accreta placentation and this histologic criterion is unlikely to be useful in confirming the diagnosis of first trimester CSEP.

Studies using surgery as a reference standard referred to a variety of different procedures. Xiong et al. [28] reported the use of intraoperative criteria during suction curettage, where it is not possible to directly visualise the CSEP. Even with surgical treatments such as hysteroscopy or laparoscopy, where visualising the CSD and implanted pregnancy is theoretically possible, maintaining optimal views can be technically challenging, especially if the uterus or pelvis is actively filling with blood. In addition, given that most CSEP cases are managed with transcervical suction evacuation [49], and not all surgical approaches allow direct visualisation of the implantation site of the pregnancy, surgical findings are not an appropriate reference standard.

The inconsistent use of terminology in CSEP research, particularly the failure to differentiate between a pregnancy on or near the scar and a true CSEP, which is implanted within the CSD, means that many studies reported cases that do not meet the recognised criteria for CSEP. Without employing standardised terminology defining a true CSEP, the comparability of studies will continue to be hindered.

### 4.4 Challenges in Establishing a Reference Standard

An ectopic pregnancy is one that implants beyond the normal boundaries of the endometrial cavity, posing a significant risk of haemorrhagic morbidity. CSEP fits this definition, but no established reference standard exists for its diagnosis. Current methods rely mainly on ultrasound to identify the anatomical placement of the pregnancy. An ideal diagnostic standard would be outcomes‐based, focusing on haemorrhagic morbidity, though this is challenging to measure. Variations in clinical presentation, diagnosis timing and treatment mean that not all CSEP cases result in significant haemorrhagic events, limiting its reliability as a diagnostic benchmark.

In the absence of a robust outcome‐based reference standard, alternative surrogate criteria are commonly employed, though these have inherent weaknesses. Ultrasound is the primary diagnostic tool for CSEP, yet it lacks a clear comparator. Diagnosis often relies on indirect criteria, such as an empty uterine cavity and an empty cervical canal. However, these criteria are transient and influenced by gestational age, reducing their reliability. The diagnostic approach for CSEP is somewhat comparable to the detection of fetal abnormalities, where ultrasound is the primary diagnostic tool, and definitive confirmation is often only possible after delivery or termination [50]. This inability to confirm the diagnosis until after termination highlights the risk of increased false‐positive diagnoses, further underscoring the difficulty in establishing ultrasound as a reference standard.

Histology is also not a robust reference standard for CSEP. While histological confirmation may be achieved in cases requiring hysterectomy, such instances are relatively rare due to the increasing trend toward timely diagnosis and conservative management. Furthermore, histological examination of excised pregnancy tissue from hysterectomy does not always reliably differentiate between CSEP and a normally implanted intrauterine pregnancy, limiting its diagnostic utility. The small proportion of cases that proceed to hysterectomy further reduces the feasibility of histology as a practical reference standard.

Given the complexities in establishing a reliable reference standard for CSEP, future research should focus on refining diagnostic criteria and exploring novel outcome‐based markers. Employing a universally applicable composite reference standard that remains valid throughout the first trimester regardless of gestational age—by combining enhanced ultrasound criteria with better‐defined clinical outcomes—may be the most effective strategy for improving diagnostic confidence and guiding clinical decisions. The key clinical implications arising from these findings for diagnosis and reporting of CSEP are summarised in Box 2.

BOX 2 **Clinical implications for practice.**

**Table:** 

Early and accurate diagnosis of caesarean scar ectopic pregnancy (CSEP) is essential to reduce maternal morbidity and preserve fertility Standardised ultrasound diagnostic criteria are needed to differentiate true CSEP (implanted within the caesarean scar defect) from low normally sited pregnancies Routine use of transvaginal ultrasound with colour Doppler imaging improves diagnostic accuracy and helps prevent false‐positive diagnoses Residual myometrial thickness and implantation site in relation to the caesarean scar defect should be documented in all cases to guide management and predict risks such as rupture or haemorrhage Agreed reporting, classification and reference standards would improve consistency across studies, support accurate diagnosis and inform future treatment guidelines

## 5 Conclusion

This scoping review found major variability in both imaging diagnostic strategies and classification criteria used to confirm CSEP at surgery and/or on histopathology. Our findings highlight the need for standardised evidence‐based study protocols and for an agreed reference standard to allow accurate comparison across imaging modalities and diagnostic markers of CSEP. We propose that ultrasound could serve as a reference standard, provided that consensus is reached on uniform diagnostic criteria to improve its reliability. Accurate diagnosis is essential for effective management of CSEP, as it directly influences management strategies, patient outcomes and use of healthcare resources.

## Author Contributions

S.N., E.J., C.B. and D.J. conceived the study. S.N. and L.V.D.B. conducted the literature search and primary analysis with review from E.J. and E.R. S.N. wrote the initial draft manuscript. All authors contributed to the interpretation of the data, revised this article critically, and agreed upon the final manuscript prior to submission.

## Funding

Dr. Simrit Nijjar's research program is supported by a scholarship from the Elizabeth Garrett Anderson Hospital Charity.

## Disclosure

Patient and Public Involvement: Patients or the public were not involved in the design, conduct, reporting or dissemination plans of our research.

## Ethics Statement

The authors have nothing to report.

## Consent

The authors have nothing to report.

## Conflicts of Interest

The authors declare no conflicts of interest.

## Supporting information


**Table S1:** Outcome reporting in CSEP trials: Search strategy.

**Table S2:** Summary of definitions and diagnostic criteria used in the studies included in the scoping review.

## Data Availability

Data available on request from authors.
