# Supplementary material for: Caesarean Scar Ectopic Pregnancy in Early Gestation: A Scoping Review of Definitions and Diagnostic Approach
Source: BJOG. 2025 Dec 25;133(5):888–99. doi: 10.1111/1471-0528.70122 (PMC12972860; doi:10.1111/1471-0528.70122)
Supplement: Supplementary file 1 — Table S1: Outcome reporting in CSEP trials: Search strategy. [file BJO-133-888-s002.docx]

**Table S1 Outcome reporting in CSEP trials: Search strategy**

| **Search PubMed/MEDLINE** | **Query** |
| --- | --- |
| #3 | #1 AND #2 |
| #2 | "Ultrasonography"[tiab] OR ultras* [tiab] OR sonohysterogra*[tiab] OR sonogra*[tiab] OR hysterosonogra*[tiab] OR echogra*[tiab] OR “transvaginal”[tiab] OR “transabdominal”[tiab] OR “three dimensional”[tiab] OR “3D”[tiab] OR “MRI”[tiab] OR “Magnetic resonance imaging”[tiab] OR “power Doppler”[tiab] OR “colour Doppler”[tiab] “color Doppler”[tiab] OR “pulsed Doppler”[tiab] OR “Doppler”[tiab] OR diagnos*[tiab] OR “definition” [tiab] OR classifi*[tiab] OR “hysteroscopy”[tiab] |
| #1 | “cesarean scar pregnancy”[tiab] OR “caesarean scar pregnancy”[tiab] OR "caesarean scar ectopic pregnancy"[tiab] OR "cesarean scar ectopic pregnancy"[tiab] OR “caesarean scar implantation"[tiab] OR “cesarean scar implantation"[tiab] OR "cesarean section scar ectopic pregnancy"[tiab] OR "caesarean section scar pregnancy"[tiab] OR "cesarean section scar pregnancy"[tiab] OR "cesarean ectopic pregnancy"[tiab] OR “intrauterine ectopic”[tiab] OR cicatrix pregnancy[tiab] OR isthmocele pregnancy[tiab] OR niche pregnancy[tiab] OR caesarean scar defect pregnancy[tiab] OR cesarean scar defect pregnancy[tiab] OR cesarean scar diverticulum pregnancy[tiab] OR caesarean scar diverticulum pregnancy[tiab] OR caesarean ectopic[tiab] OR cesarean ectopic[tiab] |
| **Search Google Scholar** | **Query** |
| #1 | *allintitle: (cesarean OR caesarean) AND scar pregnancy OR ectopic OR ultrasonography OR ultrasound OR sonography OR transvaginal OR transabdominal OR MRI OR three-dimensional OR Doppler OR diagnosis OR definition OR classification OR hysteroscopy* |
