# Supplementary material for: Caesarean Scar Ectopic Pregnancy in Early Gestation: A Scoping Review of Definitions and Diagnostic Approach
Source: BJOG. 2025 Dec 25;133(5):888–99. doi: 10.1111/1471-0528.70122 (PMC12972860; doi:10.1111/1471-0528.70122)
Supplement: Supplementary file 2 — Table S2: Summary of definitions and diagnostic criteria used in the studies included in the scoping review. [file BJO-133-888-s001.docx]

**Table S2 Summary of definitions and diagnostic criteria used in the studies included in the scoping review.**

| **Author, year** | **Defined as an ectopic pregnancy** | **Implantation site** | **CDI assessment (Y/N)** | **Placental lacunae assessment (Y/N)** | **Reported if live or failed CSEP (Y/N)** | **Diagnostic criteria (as reported in the study)** | **Classification system used** |
| --- | --- | --- | --- | --- | --- | --- | --- |
| **Wang 2013** | Y | Implanted in the scar | Y | N | Y | - Empty uterus and empty cervical canal - Development of the gestational sac or mixed-echo mass in the anterior part of the isthmic portion or in the caesarean scar - A very thin myometrium or an absence of healthy myometrium between the bladder wall and the sac or the mass, or a discontinuity in the anterior uterine wall as demonstrated on a sagittal view of the uterus when running through the amniotic sac | Type 2 (defined as deep implantation into a caesarean scar defect with infiltrating growth into the uterine myometrium and bulging from the uterine serosal surface of the uterus) |
| **Zhang 2013** | Y | Implanted in the scar | N | N | N | - No fetal parts are to be found in the uterine cavity or cervix - A thin myometrial layer between the bladder and gestational sac is present - The gestational sac is close to the bladder and uterine wall and an arteriovenous malformation is present | Type 1 (defined as gestational tissue implanted in the scar growing towards the cervicoisthmic space)  Type 2 (defined as a pregnancy mass that protruded from the uterus and grew towards the abdominal cavity) |
| **Huang 2014** | N | Implanted in the lower anterior segment | N | N | N | - Gestational sac implanted in the lower segment of the anterior uterine corpus with evidence of myometrial dehiscence, empty uterine cavity and cervical canal | N/S |
| **Liu 2014** | Y | Implanted at the scar site | N | N | Y | - No embryonic sac is seen in the uterine cavity and endocervical canal - Embryonic sac or heterogeneous masses are seen attached to the scar on the isthmus of the anterior uterine wall - The continuity of the myometrium is interrupted, and the myometrium becomes thinner at the scar - Color Doppler flow imaging (CDFI) shows obvious blood flow signals around the embryonic sac or heterogeneous masses | Gestational sac type (defined as gestational sac-like echoes seen at the incision of the lower uterine body)  Mixed mass type (defined as a mixed echo mass with unclear boundaries, uneven internal echoes, and dark areas of fluid can be seen in the scar of the lower uterine body) |
| **Timor 2014** | N | Implanted in the scar &  on the scar | Y | N | Y | - An empty uterine cavity and an empty endocervical canal - The placenta and/or a gestational sac embedded in/on a hysterotomy scar - In early gestations (<8weeks), a triangular gestational sac filling the niche of the scar; at >8weeks this shape may become rounded or even oval - A thin (1–3-mm) bladder - A closed and empty cervical canal - An embryonic/fetal pole and/or yolk sac with or without heart activity - A prominent and at times rich vascular pattern at or in the area of a Cesarean scar in the presence of a positive pregnancy test | N/S |
| **Qian 2015** | Y | Implanted in the scar | N | N | Y | - An empty uterine cavity and cervical canal - Development of the gestational sac in the anterior portion of the lower uterine segment - Absence of healthy myometrium between the bladder and the gestational sac | N/S |
| **Xiong 2016** | Y | Implanted in the scar | Y | N | N | - An empty uterine cavity and cervical canal - Development of the gestational sac in the anterior portion of the lower uterine segment - Absence of healthy myometrium between the bladder and the gestational sac | N/S |
| **Cali 2017** | Y | Implanted in the scar | Y | N | Y | - Gestational sac or trophoblast within a Cesarean section scar - Visualization of an empty uterine cavity as well as an empty endocervical canal, closed and empty cervical canal - Presence of embryonic/fetal pole and/or yolk sac with or without heart activity - Presence of a prominent and, at times, rich vascular pattern at, or in, the area of a scar in the presence of a positive pregnancy test | N/S |
| **Kaelin Agten 2017** | N | Implanted in the scar &  on the scar | Y | N | Y | - A gestational sac embedded eccentrically in the lower uterine segment - Implanted in the location of the previous CD scar - An empty uterine cavity and cervical canal - A thin or absent myometrial layer overlying the scar - The presence of a rich vascular pattern in the area of the CD scar and the placenta on Doppler ultrasound evaluation | On the scar (defined as placenta implanted partially or fully on top of a well-healed scar)  In the niche (defined as placenta implanted into a deficient or dehiscent scar) |
| **Qian 2017** | Y | Implanted in the scar | N | N | N | - Development of the gestational sac in the anterior portion of the lower uterine segment - Empty uterine cavity and cervical canal - Absence of healthy myometrium between the gestational sac and the bladder | Unruptured type of CSEP |
| **Qian, Weng 2017** | Y | Implanted in the scar | N | N | N | - Empty uterine cavity and cervical canal - Development of the gestational sac in the anterior portion of the lower uterine segment - Absence of healthy myometrium between the bladder and the gestational sac | N/S |
| **Wu 2019** | Y | Implanted in the region of the caesarean section scar | Y | N | N | - Implants in the scar of the uterine incision of the previous cesarean section - Main sonographic features include location and shape of the gestational substance, myometrium thickness in the incision region, and blood flow characteristics | Type I (defined as 1. gestational substance partially located in the incision region of the uterus, 2. irregular gestational substance shape, 3. myometrium thickness in the incision region>3mm, and 4. blood flow observed in the gestational substance located within the incision region)  Type II (defined as 1. gestational substance partially located in the incision region of the uterus, 2. irregular gestational substance shape, 3. myometrium thickness in the incision region ≤3mm, and 4. blood flow observed in the gestational substance located within the incision region)  Type III (defined as 1. gestational substance located completely within the incision region of the uterus, 2. gestational substance of irregular or regular shape, 3. myometrium thickness at the incision region ≤3mm, and  4. blood flow observed in the gestational substance within the  incision region.   - Mass type (subtype of type III, defined as cystic and solid mixed echo or solid mass, usually formed due to retained pregnancy and haemorrhage in the scar after treatment of CSEP) |
| **Li 2020** | N | Implanted at the scar site | Y | N | Y | - Empty uterine cavity: gestational sac or trophoblast mass located at the level of the previous uterine segment caesarean section scar - Thin or absent myometrial layer between bladder and gestational sac - Prominent trophoblastic/ placental circulation upon Doppler examination - Empty endocervical canal | N/S |
| **Li, Dai 2020** | Y | Implanted in the scar | Y | N | N | - Empty uterine cavity, without contact with the sac - A clearly visible empty cervical canal, without contact with the sac - The presence of the sac in the anterior uterine isthmus - An absence of or a defect in the myometrial tissue between the bladder and the sac | N/S |
| **Liu 2020** | Y | Implanted in the scar | Y | N | Y | - An empty uterine cavity and cervical canal - Detection of a gestational sac embedded in the cesarean scar - A thin or absent myometrium between the gestational sac and the bladder - The presence of abundant blood flow signals in the area of the cesarean scar | Endogenous type (defined by a fertilized egg implanting on the scar site, growing into the uterine cavity, and partially located in the lower segment of the uterine cavity)  Exogenous type (defined by a fertilized egg implanting deeply in the caesarean scar, growing in the direction of bladder and abdominal cavity, completely located in the cesarean scar) |
| **Aslan 2021** | Y | Implanted in the scar | Y | N | Y | - Visualization of an empty uterus with empty, closed cervical canal, - The gestational sac embedded in previous scar(s) - Thin or absent myometrial layer between the gestational sac and urinary bladder - Rich vascular pattern, and arteriovenous malformation around the gestational sac | N/S |
| **Shi 2021** | Y | Implanted in the scar | Y | N | Y | - No gestational sac in the uterine cavity and cervical canal - Gestational sac located at the cesarean scar in the lower uterine segment or located in the anterior wall of the uterine isthmus - Scar fissure, and interrupted or thinned myometrium of the anterior wall of the uterus in the lower uterine segment between the gestational sac and the bladder - Positive results of the “organ sliding sign”, that is, when the gestational sac was gently pressed with an US probe, the sac could slide into the cervical canal - Doppler US showed the annular blood flow signal around the gestational sac, and the pulsed Doppler showed the pulsatility index <1, and the peak velocity >20 cm/s | Type 1 (defined as partial implantation of the gestational sac)  Type 2 (defined as complete implantation of the gestational sac)  Type 3 (defined as mixed echogenic mass) |
| **Tang 2021** | Y | Implanted in the scar | Y | N | Y | - Doppler examination showing empty uterine cavity and empty cervical canal - Location of the gestational sac in the anterior part of the isthmic portion of the uterus - A thin or absent layer of the myometrium between the gestational sac and the bladder - Circular blood flow around the gestational sac | Type I (defined on the basis of the relationship between the gestational sac and myometrial thickness, >3mm)  Type II (defined on the basis of the relationship between the gestational sac and myometrial thickness, ≤3mm)  Type III (defined as gestational sac bulges out of the cesarean scar or forms an amorphous mass with rich vascularity at the cesarean scar with myometrial thickness ≤3mm) |
| **Huang 2022** | Y | Implanted at the scar site | Y | N | Y | - Gestational sac, fertilised egg and embryo are implanted at the scar of the uterine incision | Incision gestational sac type  Mass type (bulk type) |
| **Wang 2022** | Y | Implanted at the scar site | Y | N | Y | - An empty uterine cavity and cervical canal - Detection of a gestational sac embedded in the cesarean scar - A thin or absent myometrium between the gestational sac and the bladder - The presence of abundant blood flow signals in the area of the cesarean scar | N/S |
| **Cai 2023** | Y | Implanted in the scar | Y | N | Y | - In sagittal view of the uterus, a longitudinal line was drawn connecting the internal cervical os and the lower segment of the uterine cavity through the endometrium (endometrial line) as a baseline - The maximum diameter line of the gestational sac perpendicular to the central axis is shown as Line a - The widest diameter line of the cervix perpendicular to the central axis is shown as Line b - a) a<b (score 1); gestational sac morphology: ellipse (score 0) - b) a>b (score 3); Gestational sac morphology: Morphological underrule (score 1) - c): a>b (score 3); Gestational sac morphology: Morphological irregularity (score 2) - d):a>b (score 3); Gestational sac morphology: the gestational sac showed mixed echoes (score 3) | N/S |
| **Feng 2024** | N | Implanted in the scar | Y | N | N | - Type 1 - Type II - Type III | Type I (defined as part of the gestational sac tissue is implanted into the scar and grows towards uterine cavity, but most of it is implanted in the uterine cavity; the gestational sac is deformed and elongated, and the thickness of the myometrium between the bladder and the uterus is >3mm)  Type II (defined as the gestational sac is partially implanted at the scar, but most of it is implanted in the uterine cavity and may reach the bottom of the uterine cavity; the gestational sac is deformed and elongated, and the thickness of the myometrium between the bladder and the uterus is ≤ 3mm)  Type III (defined as the gestational sac is completely implanted into the scar, protruding outward to the bladder, and the myometrium between the pregnancy sac and the bladder is thinner, or even missing, with a thickness of less than 3mm) |

CDI= Colour doppler imaging; CDFI Color Doppler flow imaging; N/S, not specified.
